# Study design and protocol of a stepped wedge cluster randomized trial using a practical implementation strategy as a model for hypertension-HIV integration — the MAP-IT trial

**DOI:** 10.1186/s13012-023-01272-5

**Published:** 2023-05-10

**Authors:** Angela A. Aifah, Erinn M. Hade, Calvin Colvin, Daniel Henry, Shivani Mishra, Ashlin Rakhra, Deborah Onakomaiya, Anyiekere Ekanem, Gabriel Shedul, Geetha P. Bansal, Daphne Lew, Nafesa Kanneh, Samuel Osagie, Ememobong Udoh, Esther Okon, Juliet Iwelunmor, Angela Attah, Gbenga Ogedegbe, Dike Ojji

**Affiliations:** 1 Institute for Excellence in Health Equity, New York University (NYU) Grossman School of Medicine, New York, NY USA; 2 Department of Population Health, New York University (NYU) Grossman School of Medicine, New York, NY USA; 3 Cardiovascular Research Unit, University of Abuja Teaching Hospital, University of Abuja, Gwagwalada, Abuja, Nigeria; 4 Department of Community Medicine, Faculty of Clinical Sciences, University of Uyo, Uyo, Akwa Ibom State Nigeria; 5 Department of Family Medicine, University of Abuja Teaching Hospital, Gwagwalada, Abuja, Nigeria; 6 Fogarty International Center, NIH, Bethesda, USA; 7 Washington University in St. Louis School of Medicine, St. Louis, USA; 8 Department of Behavioral Science and Health Education, College for Public Health and Social Justice, Saint Louis University, St. Louis, USA; 9 Akwa Ibom Primary Healthcare Development Board, State Primary Health Care Development Board, Uyo, Akwa Ibom State Nigeria; 10 Department of Internal Medicine, Faculty of Clinical Sciences, College of Health Sciences, University of Abuja, Gwagwalada, Abuja, Nigeria

**Keywords:** Implementation strategy, Integrated models, NCD-HIV care

## Abstract

**Background:**

As people living with HIV (PLWH) experience earlier and more pronounced onset of noncommunicable diseases (NCDs), advancing integrated care networks and models in low-resource-high-need settings is critical. Leveraging current health system initiatives and addressing gaps in treatment for PLWH, we report our approach using a late-stage (T4) implementation research study to test the adoption and sustainability of a proven-effective implementation strategy which has been minimally applied in low-resource settings for the integration of hypertension control into HIV treatment. We detail our protocol for the *Managing Hypertension Among People Living with HIV: an Integrated Model (MAP-IT)* trial, which uses a stepped wedge cluster randomized trial (SW-CRT) design to evaluate the effectiveness of practice facilitation on the adoption of a hypertension treatment program for PLWH receiving care at primary healthcare centers (PHCs) in Akwa Ibom State, Nigeria.

**Design:**

In partnership with the Nigerian Federal Ministry of Health (FMOH) and community organizations, the MAP-IT trial takes place in 30 PHCs. The i-PARiHS framework guided pre-implementation needs assessment. The RE-AIM framework will guide post-implementation activities to evaluate the effect of practice facilitation on the adoption, implementation fidelity, and sustainability of a hypertension program, as well as blood pressure (BP) control. Using a SW-CRT design, PHCs sequentially crossover from the hypertension program only (usual care) to hypertension plus practice facilitation (experimental condition). PHCs will recruit and enroll an average of 28–32 patients to reach a maximum of 960 PLWH participants with uncontrolled hypertension who will be followed longitudinally for BP outcomes.

**Discussion:**

Given the need for integrated NCD-HIV care platforms in low-resource settings, MAP-IT will underscore the challenges and opportunities for integrating hypertension treatment into HIV care, particularly concerning adoption and sustainability. The evaluation of our integration approach will also highlight the potential impact of a health systems strengthening approach on BP control among PLWH.

**Trial registration:**

Clinicaltrials.gov (NCT05031819). Registered on 2nd September 2021.

**Supplementary Information:**

The online version contains supplementary material available at 10.1186/s13012-023-01272-5.

Contributions to the literature

- With the growing prevalence of NCDs among people living with HIV (PLWH), the HIV chronic care model offers an optimal foundation for integrating NCD programs.
- Limited studies have tested the role of an evidence-supported implementation strategy as a mechanism for integrating NCDs-HIV.
- Integrated care models for NCDs and HIV have received global calls for action as low- and middle-income countries (LMICs) are overwhelmed with comorbid diseases among PLWH.
- The MAP-IT trial leverages current initiatives and gaps in treatment for PLWH with uncontrolled hypertension in Akwa Ibom State to test the effect of an integrated NCD-HIV model on adoption, sustainability, and BP control outcomes.

## Background

With increased life expectancy, people living with HIV (PLWH) are at risk for noncommunicable diseases (NCDs) [1–3] such as cardiovascular disease (CVD) [4], for which hypertension is the primary risk factor [5, 6]. Scholars point to the successful implementation of HIV treatment programs and strategies in extending PLWH life expectancy while also emphasizing the importance of chronic or longitudinal care models [1, 5–8]. Building on calls to integrate care [2], Duffy and colleagues argue that longitudinal care models established through HIV programs offer exemplary platforms for integrating NCDs due to favorable outcomes with retention and continuity, healthy lifestyle promotion, and routine monitoring of PLWH [1]. To reduce NCDs burden in PLWH, there is an urgent need for practical implementation strategies spotlighting efficient use of available resources and strengthening healthcare delivery — especially in settings with optimal platforms or policies for integrated care and treatment.

Over the past 20 years, Nigeria, which bears the highest burden of HIV infection in Western Africa [9], experienced a dramatic increase in mortality associated with hypertension [10]. Of note, the Nigerian Federal Ministry of Health (FMOH) has a task-shifting and sharing policy that makes hypertension management integration into HIV care platforms attainable and sustainable. The policy aims to scale up access to vital health services through the efficient use of non-physician health workers to manage high mortality diseases like HIV [11]. Importantly, the policy promotes skilled, non-physician workers like nurses treating stable HIV patients and key tasks including case identification, referrals, initiation of treatment, and routine physical examinations [11]. Considering this policy, we describe below a viable HIV chronic care platform for integrating hypertension management in Akwa Ibom, a state in Nigeria with the highest HIV burden [12, 13].

The cornerstone for healthcare delivery in Akwa Ibom is at the primary care level [14]. Due to the reliance on non-physician health workers to deliver primary services to PLWH, primary healthcare centers (PHCs) with HIV programs are strategic platforms for implementing hypertension management. The Akwa Ibom State Primary Healthcare Development Agency (hereinafter “the Agency”) oversees 425 PHCs to strengthen primary healthcare delivery, including HIV/AIDS response in 31 local government areas (i.e., districts) [14]. The Agency supports access to affordable, sustainable, and equitable primary healthcare and HIV services through partnership, resource mobilization, development of community-based systems, and functional primary healthcare infrastructure [15]. Although PHCs in Akwa Ibom provide effective treatment for PLWH, integration of evidence-based hypertension management strategies within these settings is untested.

Our NIH-funded project, *Managing Hypertension Among People Living with HIV: an Integrated Model (MAP-IT)*, tests an innovative approach to integrate hypertension care for PLWH, with emphasis on the adoption and sustainability of an evidence-based hypertension management program, using an implementation strategy. We aim to evaluate the effectiveness of practice facilitation (PF) on the integration of a task-strengthening strategy for hypertension control (TASSH) in PHCs in Akwa Ibom. While similar to an ongoing study in Lagos [16], this study is conducted in collaboration with the Agency and features several distinctions, notably the implementation science framework and study design applied. Below, we (1) provide an overview of the hypertension program and implementation strategy, (2) discuss the implementation science frameworks used, and (3) detail the study design and analysis for this implementation science protocol.

### Overview of the hypertension program and implementation strategy

Two key components define our protocol: a hypertension management program and an external support system for nurses. A nurse-led (TASSH) program will be implemented at all PHCs. TASSH includes identification of patients with hypertension, treatment of patients with uncontrolled hypertension using antihypertensive medication based on a standard treatment and drug titration protocol (see Supplemental file A), lifestyle counseling, and referral to physicians for complicated cases of hypertension (i.e., nonresponsive to first-line treatment after maximum doses, *BP* ≥ 180/110 mm Hg, or with comorbid diabetes mellitus, heart attack, stroke, kidney disease, or heart failure, or pregnancy following study enrollment) [17]. With the exception of TASSH focusing on nurses providing hypertension care, these components of TASSH are similar to the Nigerian Hypertension Treatment Protocol. The experimental implementation component, PF, is an approach to service integration that will be introduced to all PHCs in a stepwise manner over the duration of the trial. PF involves provision of external expertise on practice redesign and a tailored approach to evidence-based care [18–22]. It includes both a role (practice facilitator) and a process for supporting primary care practices to build motivation and capacity at the systems and individual levels to improve organizational performance [19, 21–24]. Practice facilitators (typically nurses) are trained to work with primary care practices to develop the necessary skills to adapt evidence-based strategies, e.g., task shifting/sharing, to their practice environment [25]. In this study, the practice facilitators are nurses with an average of 35 years in clinical practice.

## Methods

### Study design

**Fig. 1:**
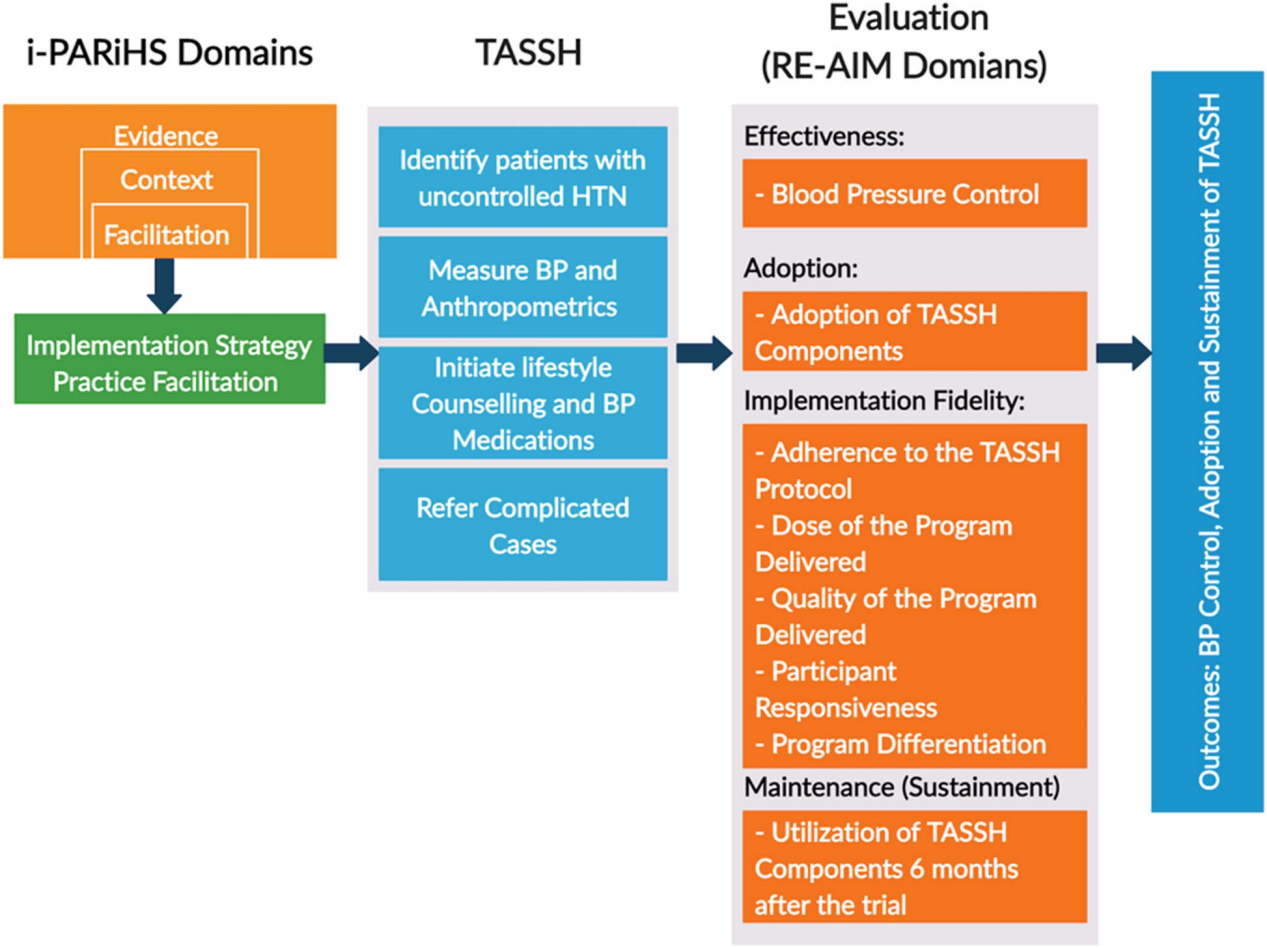
Overview of i-PARiHS and RE-AIM

This study occurs in multiple phases [a pre-implementation (UG3), implementation (UH3), and post-implementation], applies several implementation science frameworks, and uses a stepped wedge cluster randomized trial (CRT) study design. Figure 1 overviews the two implementation science frameworks — the integrated Promoting Action on Research Implementation in Health Services (i-PARiHS) [26] and the Reach, Effectiveness, Adoption, Implementation, and Maintenance (RE-AIM) [27] — used in the pre- and post-implementation phases. Below, we provide details on each phase.

The pre-implementation phase used the i-PARiHS in inquiring about the existing health system and PHC context to inform the development of a context-specific PF strategy. We assessed barriers and facilitators of integrating TASSH into PHCs for hypertension management through structured facility assessments and in-depth interviews with stakeholders within the PHCs and primary health system in Akwa Ibom. A pilot study was conducted during the pre-implementation phase wherein the feasibility of TASSH plus PF intervention was assessed in two PHCs. The facilities included in the pilot are not participating in the implementation phase.

**Fig. 2:**
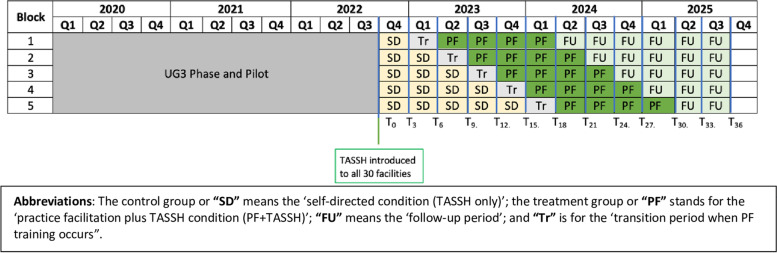
Stepped wedge study design

In the implementation phase, we are using a SW-CRT design to introduce PF at the PHCs. The 30 PHCs were randomly allocated into five cohorts of six PHCs, and all begin in the TASSH only condition, which serves as the usual care control. Figure 2 overviews the stepped wedge design for this study. Given the SW-CRT, the length of time in TASSH alone (without PF) will vary, with a minimum duration of 3 months for the first cohort. Three months prior to the TASSH plus PF experimental condition starting, each cohort will have a training period. During this period, nurses and other staff at the PHCs receive training (or retraining) in delivering TASSH. Once the 3-month training period is completed, the PHCs will participate in a 12-month implementation period during which PF will occur.

Nurses at these PHCs will implement all aspects of TASSH in their clinical care for PLWH with uncontrolled hypertension. PFs will support nurses during the 12-month period by helping with hypertension treatment issues and addressing barriers to TASSH implementation. The RE-AIM framework defines measures to evaluate the effect of PF on TASSH adoption after 12 months of PF compared to TASSH adoption without PF (primary outcome). RE-AIM facilitates evaluation of the effect of PF on BP control (secondary outcome) and the level of TASSH implementation fidelity (secondary outcome).

**Fig. 3:**
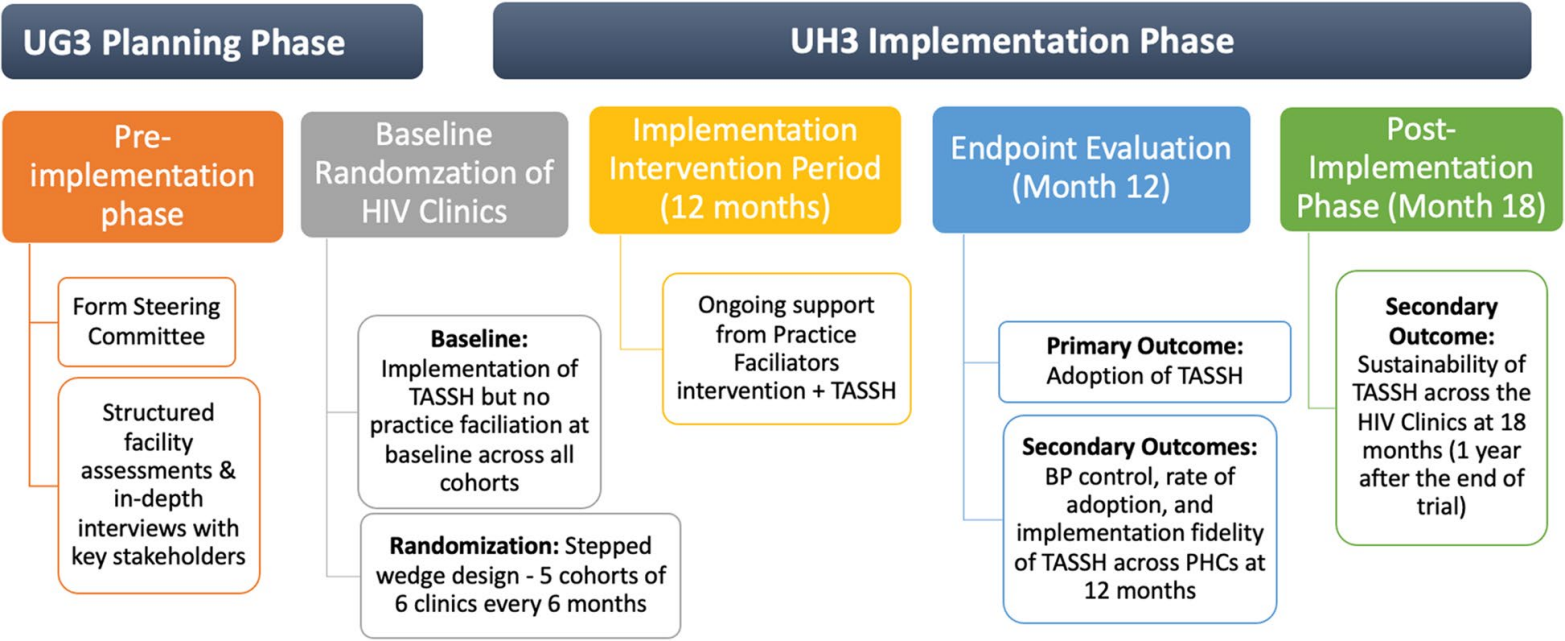
Pre-implementation (UG3), implementation (UH3), and post-implementation phases

Following each cohort’s 12-month implementation phase, each PHC transitions to the post-implementation phase (follow-up period). Nurses will continue to implement TASSH. However, PF will end. Six months into this post-implementation period (18 months following the start of PF), we will evaluate the sustainability of TASSH within the PHCs (secondary outcome). Figure 3 provides an overview of the study design including the pre-implementation, implementation, and post-implementation phases.

### Ethical approvals

This study is approved by the Institutional Review Boards (IRBs) at the University of Abuja (Abuja, Nigeria), Ibom Multi-Specialty Hospital (Uyo, Akwa Ibom), and New York University (NYU) Grossman School of Medicine. The study is registered at Clinicaltrials.gov (NCT05031819).

### Rationale for the study design

In a pragmatic study design which stems from the fields of service delivery and policy evaluation, the SW-CRT allows for robust evaluation while factoring in logistical (i.e., rolling out of facilities) or political (i.e., relating to the policymakers and service managers) constraints common with a standard parallel randomized controlled trial (RCT) design [28]. In this study, the sequential crossover of PHCs from TASSH-only to TASSH-plus PF permits rigorous evaluation of the implementation strategy effects while enabling all study facilities and participants the benefit of TASSH with PF training.

Along with the robust nature of the stepped wedge design, we will evaluate the PF experimental condition using the RE-AIM framework. Widely used in both health research and health intervention programming and evaluation, RE-AIM offers a systematic guide for collecting and analyzing data to examine all aspects of planning, implementation, and potential sustainability [29]. For this study, RE-AIM is most appropriate to evaluate and identify the program elements critical for implementation and sustainability.

### Study setting and population

Thirty-two PHCs from a network of 59 PHCs across Akwa Ibom were recruited into the study — 2 for the pre-implementation phase and 30 for the implementation phase. These PHCs support a sizeable number of PLWH. Adoption will be measured on all eligible persons to be screened for hypertension. Eligible patients are identified from the roster of PLWH during routine visits and through linkages from community screening and proximal HIV clinics. In the 30 implementation study sites, we will recruit and enroll an average of 28–32 patients, for a maximum of 960 PLWH participants with uncontrolled hypertension to be followed longitudinally for BP outcomes. Eligibility criteria include the following: (1) HIV-positive persons aged 18 years or older; (2) either currently enrolled in HIV treatment services at one of the 30 study PHCs, receiving hypertension treatment services at one of the 30 study PHCs, or can be enrolled at one of the 30 study PHCs to receive hypertension services for the entire study duration; (3) (for those being followed for BP control) have elevated BP between 140–179 mm Hg systolic and/or 90–109 mm Hg diastolic, as determined by the average of the latter two of three separate BP readings during one clinic visit; and (4) be able to provide consent. The inclusion criteria are based on WHO’s CVD treatment guidelines [30–32].

Those who refuse, who are unable to provide informed consent, or with any of the following criteria at baseline will be ineligible for inclusion in the longitudinal BP cohort: (1) BP of ≥ 180/110 mm Hg; (2) known history of kidney disease, heart disease, diabetes mellitus, transient ischemic attacks, stroke, or heart failure; and (3) are pregnant or breastfeeding at the time of enrollment.

### Description of TASSH and the PF implementation strategy

As previously noted, our Lagos study [16] provides thorough details of TASSH and the approach for developing the PF implementation strategy. Here, we briefly describe both and specify any potential differences for this study.

TASSH uses a four-step approach for identifying, counseling, treating, and referring (ICTR) and the 5As counseling strategy (ask, assess, advice, assist, & arrange). The four steps are defined as follows: (1) identifying PLWH patients with uncontrolled hypertension (*BP* > 140/90); (2) initiating counseling by giving clear advice on how PLWH patients can modify their behaviors; (3) treating PLWH with uncontrolled hypertension by prescribing medication treatment according to the Nigerian Simplified Hypertension Protocol; and (4) referring patients with severely elevated BP and/or a chronic comorbid condition, including established CVD, to physicians for further care. The 5 As have been implemented in previous TASSH studies [11, 16, 17, 25] and applied to other health outcomes [33, 34].

As described, we will test the effect of PF on the adoption of TASSH and systolic BP reduction. We used a context-specific approach to develop and guide the PF implementation strategy based on three main components: (1) formation of an advisory board to guide the PF intervention, (2) development of the PF strategy, and (3) training of community nurses on TASSH protocol implementation.

The advisory board constitutes members from the National Agency for the Control of AIDS (NACA), the Federal Ministry of Health (FMOH), the Directorate of Nursing, Akwa Ibom State Ministry of Health, Akwa Ibom State Agency for Control of AIDS (AKSACA), two physicians, two community nurses, and three patient advocates. Development of the PF strategy included the following: (1) training the practice facilitators using a train-the-trainer model [35] guided by the study-developed three Es, i.e., engaging, enhancing, and evaluating the nurses on performing their TASSH duties and delivering the hypertension protocol, (2) identifying site champions and coordinators, (3) building consensus for quality improvement targets, (4) implementing practice changes to support TASSH implementation, and (5) peer-to-peer collaboration. Additional details on the training approach for TASSH and the PF implementation strategy are detailed in prior work [16].

### PF justification and approach

PF is designed to stimulate specific, actionable steps that PHCs can use to build a robust and context-appropriate foundation for integrating TASSH components. Senior or retired healthcare nurses are trained as coaches and help develop the skills needed in order to adopt evidence-based interventions. The PF strategy combines one-on-one onsite facilitation with shared learning opportunities across practices via peer-to-peer phone calls and text messaging. Practice facilitators are expected as follows: (i) coordinate meetings with PHC nurses, (ii) assist PHC nurses in setting performance goals, (iii) strategize on how to best implement TASSH, and (iv) test workflow changes within PHCs.

During the implementation phase, practice facilitators will support PHCs and nurses implementing TASSH. Facilitators will coordinate monthly telephone calls and onsite meetings (twice during the first month and monthly thereafter). Key tasks for the onsite meetings include reviewing baseline data and assisting nurses to set targets for their weekly workload at the first meeting and troubleshooting workflow issues in subsequent visits. Monthly check-in phone calls will provide opportunities for nurses to raise challenges that may arise with implementing TASSH.

### Enrollment, randomization, and allocation

In the pilot phase, two PHCs were recruited to refine and understand data collection strategies, instruments, training timing and methods, and study recruitment methods. For the SW-CRT, 30 PHCs are randomized in five cohorts of six PHCs. Within each PHC, we anticipate enrolling approximately 28–32 PLWH on average. The enrollment targets for each PHC are based on its smaller, mid-sized, or large caseload. We do not anticipate any attrition in study sites. In the event that a site does drop out for any reason, we have adequate numbers of sites with similar characteristics to recruit from PHCs that the Agency oversees.

Study biostatisticians developed the randomization sequence to allocate each PHC to a stepped wedge cohorts. These allocations are kept in a secure electronic format inaccessible to study sites, in accordance with CONSORT guidelines. Study coordinators will be informed of which PHCs are allocated to each cohort just before each training period begins. Because of the nature of the intervention, it is impossible to mask individuals, nurses, practice facilitators, and study coordinators to the group assignment when implementation begins. One study biostatistician will remain blinded to treatment assignment until all data have been collected and the database is locked.

### Recruitment and retention

#### Primary healthcare centers (PHCs)

Throughout Akwa Ibom, the majority of PHCs provide HIV treatment services. Building on the pre-implementation phase findings, we worked closely with the Akwa Ibom State Ministry of Health and the Agency to select two pilot PHC sites and the 30 PHCs for the SW-CRT. Selection of eligible PHCs was based on geographical areas distinct from one another to allow for an equal mix of urban and rural PHCs. PHCs agreed to participate, signed memorandum of understanding (MOU) agreements, and identified at least two nurses to be trained to deliver TASSH and collect study data. Leveraging our experience from our other trials [17, 25, 36] and best practices from the literature on PF [21, 37, 38], we will use several strategies to retain PHCs enrolled in the study. These include the following: signed MOUs, identifying a champion or facility case manager as a liaison, providing monetary incentives for nurses, and maintaining open communication through phone calls, emails, etc.

#### Patients

All PLWH treated at the PHCs will receive TASSH, as it is aligned with the Nigerian Hypertension Treatment Protocol, the current standard of care. Working closely with study trained nurses at the 30 PHCs and based on the data from the facility assessment, research coordinators will develop a recruitment strategy tailored to each study site for the BP longitudinal cohort. After study trained nurses identify an eligible PLWH with uncontrolled hypertension meeting the inclusion criteria, he or she will describe the study and refer the patient to a research coordinator to obtain informed consent if the patient is willing to participate. Each PHC will recruit an average 28–32 PLWH with uncontrolled hypertension who meet the eligibility criteria for the BP longitudinal cohort.

#### Practice facilitators

Senior nurses with extensive clinical and managerial experience working in PHCs as trainers were recruited as practice facilitators to support clinic nurses implementing TASSH. Working with the Akwa Ibom State Directorate of Nursing which oversees the training, planning, and evaluation of capacity building programs, we recruited and hired six senior nurses as practice facilitators. Each practice facilitator will work with six study sites for the study duration. The decision to recruit and hire senior nurses as practice facilitators is due to the hierarchical nursing structure in Nigeria wherein senior nurses are responsible for providing oversight and support of nurses within PHCs. Working within the current nursing structure enhances the sustainability and adoption of TASSH beyond the study period.

### Primary outcome

Adoption (i.e., uptake) is the utilization of components of TASSH. TASSH adoption will be defined as nurses showing utilization of all of the following components: (1) identifying PLWH with uncontrolled hypertension, (2) measuring PLWH’s anthropometrics and BP with a valid automated device following standard procedures, (3) initiating lifestyle counseling and medication treatment every 1–3 months, and (4) referring PLWH with severely elevated BP and complicated hypertension to physicians for further care. Adoption will be measured at the clinic level, in aggregate, each month from the baseline period of the first cohort through the end of follow-up, for all those PLWH with BP screens by case managers or those previously diagnosed with hypertension at a prior visit by the nurses. Table 1 expands on the specific measures of adoption.

**Table 1:** Measurement of adoption

Outcome	Components	Data sources	Measurement
**Adoption**	**Identifying PLWH with uncontrolled hypertension**		
	• Measuring blood pressure • Measuring anthropometrics • Assessing comorbidities • Asking about lifestyle factors (physical activity, alcohol consumption, tobacco use, and diet)	Blood pressure monitoring register Hypertension treatment card	Proportion of PLWH who receive all applicable components of the protocol at each PHC during each time period
	**Counseling PLWH on lifestyle factors**		Adoption will be measured each month at each clinic utilizing aggregate data
	**Treating and referring** • Initiating treatment under the Nigeria hypertension protocol • Titrating drugs under the Nigeria hypertension protocol • Referring PLWH with complicated hypertension to secondary health center (as needed)		

### Secondary outcomes

Secondary outcomes include BP control, implementation fidelity, and TASSH sustainability. BP control is defined as a BP less than 140/90 mm Hg. For those consented and enrolled, at baseline visit, three BP readings will be taken by nurses using an automated BP monitor with the participant seated comfortably for 5 min prior to the measurements, following standard guidelines. The average of the latter two BP readings will be used as the measure for each visit. The same procedure will be followed for each participant visit, including the 12-month visit. Uncontrolled BP is defined by an average clinic systolic *BP* ≥ 140 mm Hg or diastolic *BP* ≥ 90 mm Hg following the guidelines set forth by WHO for CVD treatment [31, 32] and in accordance with the Nigeria Hypertension Treatment Protocol.

Fidelity to TASSH will be measured throughout the study and defined by five dimensions: (1) adherence to the program protocol, (2) dose of the program delivered, (3) quality of program delivery, (4) participant responsiveness, and (5) program differentiation. Tools to assess fidelity were developed for this study and adapted from the current literature [39–41]. Fidelity measures will evaluate adherence from the perspective of the PF, nurses, and the participants.

Sustainability is defined as the maintenance of TASSH adoption at HIV clinics at 18 months (6 months after the end of each cohort’s TASSH + PF phase). Sustainability will be assessed similarly to adoption (as defined above) and qualitatively, based on interviews with nurses and clinic leadership at 18 months. The research coordinator will conduct the interviews with two nurses and one key leadership personnel at each clinic. The interviews will be guided by i-PARiHS and inquire about attitudes regarding TASSH implementation, barriers, facilitators, and implications for scalability. All interviews will be recorded, transcribed, and analyzed with NVivo Version 11.

## Statistical approach

### Power and sample size justification

As the level of randomization for this SW-CRT is at the practice level, there are several important study design features: *K*, the number of primary care practices, and *m*, the average number of PLWH recruited per practice, as well as the relatedness of individuals within sites (when individual level data are utilized for analysis). Specifically, the *m* participants will be nested (grouped) within the *K* practices. This nesting will lead to additional dependency in outcomes of participants within a practice; the degree of this dependency, the intraclass correlation coefficient (ICC), will contribute to the necessary increase in sample size to maintain the same level of power as would result if the unit of randomization was independent individual participants rather than participants within practices. Prior studies examining the effects of TASSH on BP control have demonstrated improvements in BP control level ranging from 20 to 25% over usual care. We expect a somewhat smaller effect, as we are testing the effect of PF plus TASSH on BP control.

Trials targeting BP control have found ICCs for BP control ranging between 0.02 and 0.06. In addition, preliminary evidence from our UG3 pilot phase suggests that ICC estimates for BP control under UC align with these estimates. Adoption will be measured as outlined above through aggregate data at the site level each month. It is expected that TASSH adoption will be lower than 50% before PF. We expect the estimates in Table 2 to be conservative if the baseline proportion for adoption is less than 40%. We will test our primary and secondary hypotheses at the 5% two-sided type 1 error level. For secondary measures that will utilize individual level data, we expect to enroll 28–32 individuals on average, expecting approximately 10% attrition over the course of the study period, resulting in at least 25 on average per site.

**Table 2:** Achieved power for differences in outcomes ranging from 15 to 20%

ICC	At baseline (no PF): 50%		At baseline (no PF): 40%	
	**Difference: 15%** **Odds ratio: 1.9**	**Difference: 20%** **Odds ratio: 2.3**	**Difference: 15%** **Odds ratio: 1.8**	**Difference: 20%** **Odds ratio: 2.2**
**0.01**	**82%**	**97%**	**84%**	**98%**
**0.03**	**79%**	**96%**	**80%**	**96%**
**0.05**	**77%**	**95%**	**78%**	**96%**

Assume 5% two-sided type 1 error, *K* = 30, *m* = 25 (after accounting for 10% attrition, *m* = 28 at time of recruitment)

### Primary outcomes analyses

TASSH adoption will be measured and evaluated as described above, on all adult PLWH who access care through or visit the sites. Aggregate, de-identified data will be collected from sites each month (cross-sectional measures) to assess adoption. To examine the difference in the proportion of individuals indicated as meeting adoption criteria following 12 months of PF compared to adoption during UC, we will utilize a generalized linear mixed model (GLMM). A GLMM regression model will include a fixed effect for time/step interval, a fixed effect for treatment group at time *t*, and random effects for PHC/cluster. We will explore alternatives to these models in sensitivity analyses outlined below.

We will classify four intervals of the PF intervention, each 3 months in duration (start of PF + TASSH to 3 months; 4 to 6 months; 7 to 9 months; 10 to 12 months) to explore the change in adoption over the intervention period. Adoption at the cluster/site level will be described and compared across intervals. To examine whether the rate of adoption increases over time, a GLMM will be used to assess the effect of time on adoption rates. Specifying a main effect of time (categorized as defined above), we will interpret a positive main effect to indicate that rates of adoption increase over time. Covariates strongly related to adoption as well as study design factors which may influence levels of adoption will be identified and may be included in adjusted analyses. Proposed factors include site size and patient characteristics such as sex and age (as a proportion of the total). These will be a limited set of covariates, given the limited degrees of freedom available for these site/cluster-level analyses. Adjustment variables will be identified prior to data exploration and PF initiation. The denominator degrees of freedom for the *F*-test for the intervention effect contrast will be adjusted for the number of clusters and will consider any group level covariates utilized in adjustment [42]. The test of PF intervention to usual care will be evaluated at the two-sided 5% level.

### Secondary outcomes analyses

#### BP control

As noted previously, controlled BP will be defined as systolic blood pressure (SBP) less than 140 mm Hg and diastolic blood pressure (DBP) less than 90 mm Hg. In a closed cohort analysis (i.e., restricted to only enrolled PLWH), we will examine the improvement in BP control on those enrolled during the initial months of the trial. To examine the difference in the proportion of enrolled individuals with BP control during the PF of TASSH to the UC intervals following 12 months of PF, we will use GLMM to assess the intervention effect. A GLMM regression model will include a fixed effect for time interval, a fixed effect for treatment group at time *t*, and random effects for cluster, for individuals within clusters, and for time within cluster. These random effects will accommodate the dependence between outcomes assessed in the same participant over time, as well as for the correlation of individuals within sites. Selected individual-level covariates highly related to BP control will be included for adjustment, including biologic sex. Cluster-level covariates may also be considered for adjustment if possible, such as clinic size. The denominator degrees of freedom for the *F*-test for the intervention effect contrast will take into account any group-level covariates utilized in adjustment.

#### Implementation fidelity

We will assess implementation fidelity of TASSH as a potential mechanism that may explain the effect of PF on our primary outcome of BP control. Analyses will begin with descriptive analyses of levels of TASSH implementation fidelity. Using validated surveys, we will also assess practice-level, provider-level, and patient-level moderators that may influence implementation fidelity of TASSH within the PHCs. Finally, we will examine the association between the proportion of BP control at 12 months following PF initiation and levels of implementation fidelity for TASSH. Quantitative analysis of implementation fidelity will consist of descriptive statistics to provide documentation and description of fidelity to each of the components of TASSH. Associations between BP control and implementation fidelity will be evaluated using both the methods outlined by Vanderweele [43, 44], along with structural equation modeling (SEM) methods. We will estimate a path model to investigate relationships between BP control and implementation fidelity. Implementation fidelity will be identified as individual and simultaneous predictors of BP control at the site level. Practice-level, provider-level, and patient-level variables, which may influence implementation fidelity, will be considered in the model. In addition to the direct effects of each variable, the indirect effects from each variable to BP control via mediator variables will be estimated. This analysis will examine potential pathways in which BP control is influenced by fidelity to TASSH and characteristics of practices, providers, and PLWH in the trial [45]. Fit indices will be evaluated for all path models to ensure adequate model fit.

#### Sustainability

Sustainability of TASSH will be evaluated 6 months after the completion of the PF implementation interval. Sustainability will be defined in the same manner as adoption and classified using two 3-month intervals during the follow-up measurement period lasting 6 months. Rates of sustainability will be represented using descriptive statistics (rates of sustainability overall and by clinic). Levels of sustainability over time will be represented with descriptive tables. We will compare sustainability of TASSH to adoption in the intervention interval through descriptive summaries across sites. To examine whether sustainability changes over time, we will use a GLMM to assess the effect of time on sustainability. Specifying a main effect of time with sustainability as our outcome, we will interpret a main effect of negative magnitude to indicate that rates of sustainability decrease over time. We hypothesize that sustainability will not decline over time. Potential covariates and confounding factors which may influence levels of sustainability will be identified and may be included in adjusted analyses, if possible, due to limited degrees of freedom for the sites/clusters.

### Handling missing data

Maximum likelihood estimation of mixed-effects models will be used to account for missing data. In the context of a mixed-effects model, this is equivalent to an assumption of data missing at random. We will conduct sensitivity analyses assessing this assumption including comparison of participants with and without missing values with respect to baseline and practice characteristics. If differential patterns emerge, we will consider the use of multiple imputation by chained equations and/or inverse probability weighting to adjust for missing data.

### Sensitivity analysis

While primary analyses will focus on estimation through GLMM and take all information gathered during the usual care and PF periods into account, we will also consider the evaluation of PF during the “active stepped wedge” phase, the period in which at least one block differs in terms of treatment group (those periods when all blocks are in usual care or PF are excluded). These evaluations will compare estimates of the GLMM method and those which estimate the treatment effect through robust nonparametric permutation methods. GLMMs require assumptions on the random effects, which if violated result in biased treatment effect estimates [46, 47]. The proposed permutation tests do not require these assumptions on the correlation structure but require that all clusters are not in the same treatment condition. We will assess the robustness of GLMM assumptions through the comparison of results from methods in this reduced set of observations.

## Discussion

For low-resource-high-need settings, overwhelmed by suboptimal healthcare systems and healthcare workforce shortage, context-specific approaches for integrating effective, evidence-based approaches are critically needed [16, 25]. Our study, MAP-IT, is one of few implementation science studies to include a health services’ strengthening implementation strategy approach to integrate hypertension management into HIV care and treatment in Akwa Ibom State, Nigeria, which has one of the highest HIV prevalence rates in the country. Specifically, the study is using an evidence-based task strengthening hypertension program and PF as the implementation strategy to enhance the adoption and sustainability of hypertension control and management among PLWH and their providers. Our PF strategy builds on current knowledge regarding how facilitation functions by the following: tailoring the hierarchical structure within PHCs to work with senior or retired nurses as facilitators focusing primarily on supporting HIV nurses to treat hypertension, enhancing clinic workflow by troubleshooting and goal setting with HIV nurses, and reinforcing the current hypertension treatment protocol in Nigeria as the standard of care for PLWH.

MAP-IT is one of the first studies to apply the i-PARiHS framework to the African context, as part of pre-implementation activities, to assess the capacity of healthcare facilities to deliver an evidence-based hypertension control program. The testing of TASSH among PLWH is also relevant given the recent implementation of the Nigerian Hypertension Treatment Protocol as the standard of care for treating individuals with hypertension at the primary care level. Previous studies demonstrate that primary care practices are more likely to adopt evidence-based standards of care through PF [18, 20, 22]. It is not clear, however, that this conclusion applies to PHCs in low- and middle-income countries (LMICs) experiencing a dual disease burden of chronic NCDs and HIV. MAP-IT will advance research and practice on the potential impact of PF as an implementation strategy to improve the adoption of hypertension standard of care for PLWH while providing health system-level improvements and outcomes in an LMIC setting.

More precisely, this study will provide evidence on the implications of engaging senior or retired healthcare workers as practice facilitators on the adoption and sustainability of an evidence-based hypertension control program for PLWH. In a country where these individuals are usually retired early at the age of 60 years or after 35 years of practice, adopting this strategy will help tap into their rich expertise. In short, our assessment of PF will provide key evidence on the impact of external facilitators on hypertension outcomes among PLWH and as a potential health services’ strengthening approach to support non-physician providers in LMICs experiencing a growing prevalence of comorbid HIV and hypertension.

## Limitations

Given the stepped wedge study design, there are methodological challenges to consider for this study. Despite the benefits of the stepped wedge design, a notable limitation is potential confounding due to temporal trends [28], wherein more clusters/PHCs are exposed to PF as the study progresses as opposed to the beginning. This scaffolding of PF will limit our ability to disentangle effects of time from the PF implementation strategy, i.e., the first PHCs/cohorts will be exposed to PF longer than later PHCs. To address this limitation, randomization of PHCs along with robust adjustment for temporal trends in modeling will be key.

## Implications

The results of our study will provide evidence for scaling up TASSH as a standard approach to reduce the burden of uncontrolled hypertension in PLWH. Furthermore, our understanding of PF as an effective strategy to improve the adoption of evidence-based practices in clinical settings remains incomplete because previous work has not examined PF in LMIC settings experiencing increased hypertension and HIV comorbidity. Lastly, the MAP-IT study is timely in its implementation, and the potential implications of the findings on strengthening integrated NCD-HIV care programs as PLWH are living longer and are increasingly at risk of having comorbid NCDs.

## Supplementary Information

**Additional file 1.** Nigeria Hypertension Treatment Protocol (https://linkscommunity.org/assets/PDFs/nigeria-hypertension-protocol-04.pdf)

## Data Availability

Not applicable.

## Abbreviations

AKSACA: Akwa Ibom State Agency for Control of AIDS
BP: Blood pressure
CRT: Cluster randomized trial
CVD: Cardiovascular disease
DBP: Diastolic blood pressure
GLMM: Generalized linear mixed model
HIV: Human immunodeficiency virus
ICTR: Identifying, counseling, treating, and referring
FMOH: Federal Ministry of Health
i-PARiHS: Integrated Promoting Action on Research Implementation in Health Services
IRB: Institutional review board
LMICs: Low- and middle-income countries
MAP-IT: *Managing Hypertension* *Among* *People Living with HIV: an Integrated Model*
MOU: Memorandum of understanding
NACA: National Agency for the Control of AIDS
NCDs: Noncommunicable diseases
PF: Practice facilitation
PLWH: People living with HIV
PHCs: Primary healthcare centers
RE-AIM: Reach, effectiveness, adoption, implementation, and maintenance
SEM: Structural equation modeling
SBP: Systolic blood pressure
TASSH: Task-strengthening strategy for hypertension control
UNAIDS: Joint United Nations Programme on HIV/AIDS
WHO: World Health Organization
