# Supplementary material for: Study design and protocol of a stepped wedge cluster randomized trial using a practical implementation strategy as a model for hypertension-HIV integration — the MAP-IT trial
Source: Implement Sci. 2023 May 10;18:14. doi: 10.1186/s13012-023-01272-5 (PMC10173657; doi:10.1186/s13012-023-01272-5)

**Supplemental 1 Appendix:** **Nigeria Hypertension Treatment Protocol** (<https://linkscommunity.org/assets/PDFs/nigeria-hypertension-protocol-04.pdf>)


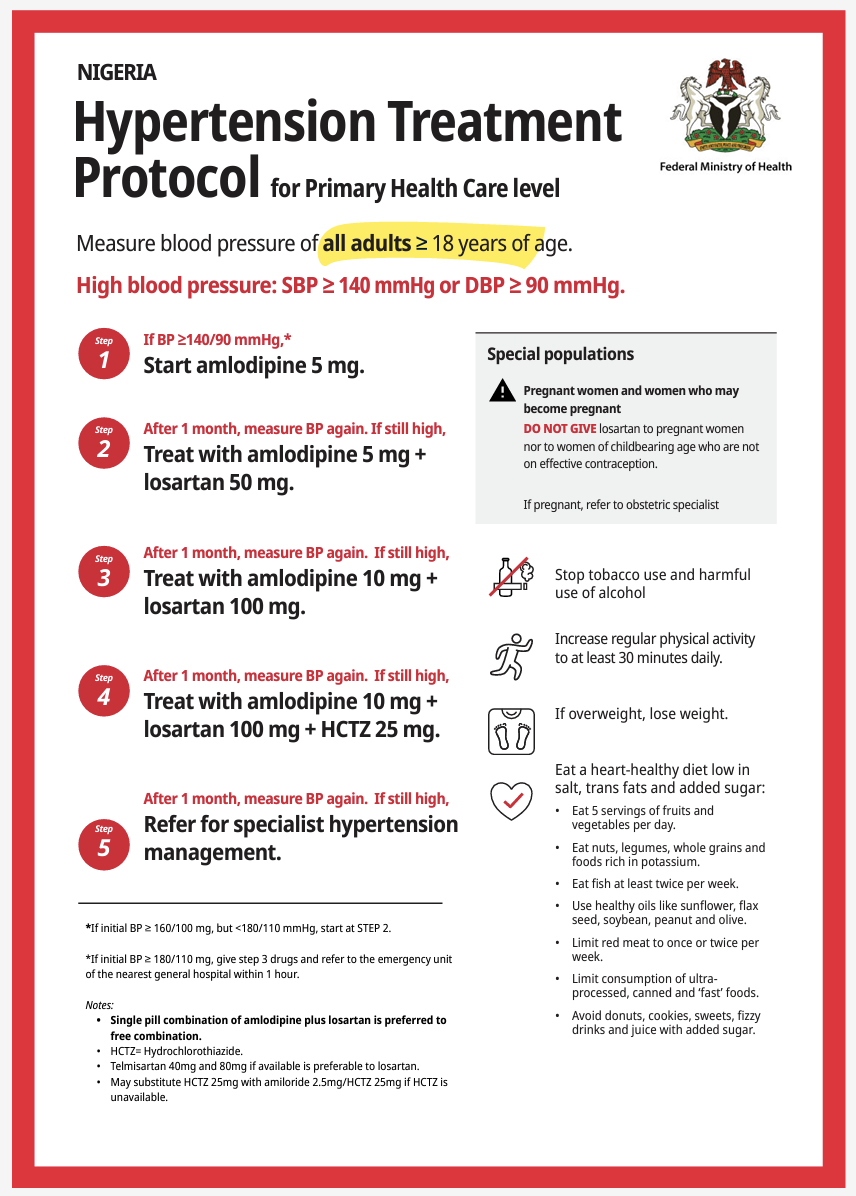

Supplement: Supplementary file 1 — Additional file 1. Nigeria Hypertension Treatment Protocol (https://linkscommunity.org/assets/PDFs/nigeria-hypertension-protocol-04.pdf) [file 13012_2023_1272_MOESM1_ESM.docx]
